# Development, Validation, and Implementation of a Geography-Specific Syndromic Coding and Reporting System for Snakebite Envenoming (D-SERPENT): Protocol for an Implementation-Effectiveness Hybrid Study

**DOI:** 10.2196/98861

**Published:** 2026-11-10

**Authors:** Srikanta Kanungo, Omesh Kumar Bharti, Surajit Garabadu, Rahul K Gajbhiye, Joseph K Joseph, Rajesh Kumar Sood, Kapil Aher, Swagatika Pati, Hrishikesh Munshi, Bipin Thakur, Subrata Kumar Palo, Sabarish B Nair, Sumit Aggarwal, Amitava Banerjee, Jaideep C Menon

**Affiliations:** 1 ICMR-RMRCBB, NALCO Nagar, Chandrasekharpur Bhubaneswar, Regional Medical Research Centre, NALCO Square, Chandrasekharpur, Bhubaneswar, ODISHA, 751023, India; 2 Department of Health & Family Welfare, Government of Himachal Pradesh, Himachal Pradesh, India; 3 Department of Health & Family Welfare, Government of Odisha, Bhubaneswar, Odisha, India; 4 ICMR-National Institute for Research in Reproductive and Child Health, Mumbai, Maharashtra, India; 5 Little Flower Hospital & Research Centre, Angamaly, Kerala, India; 6 Medical Officer Health, Kangra, Himachal Pradesh, India; 7 Public Health Department, Deputy Director of Health Services, Nasik Division, Government of Maharashtra, Nashik, Maharashtra, India; 8 Chief Medical Officer Office, Chamba, Himachal Pradesh, India; 9 Amrita Institute of Medical Sciences, Amrita Vishwa Vidyapeetham, Amrita Lane, Elamakkara P.O. Ponekkara, Kochi, Kerala, 682026, India, 04842802000; 10 Indian Council of Medical Research, New Delhi, India; 11 Institute of Health Informatics, University of London, London, United Kingdom

**Keywords:** venomous snake, coding for disease, syndromic approach, hemotoxic, neurotoxic, disability-adjusted life year, DALY, snakebite envenoming, SBE

## Abstract

**Background:**

Snakebite envenoming (SBE) is a neglected tropical disease with substantial morbidity and mortality. India accounts for nearly 50% of global snakebite deaths, yet the disease remains underreported due to a lack of standardized documentation and phenotypic classification. Current diagnosis is syndromic and lacks uniform data capture protocols.

**Objective:**

This study aims to develop, validate, and implement a geography-specific syndromic coding and reporting system for SBE to improve standardized documentation, clinical management, surveillance, and epidemiological data quality in selected districts of India.

**Methods:**

This implementation-effectiveness hybrid study will be conducted across 4 study districts in India, representing 4 geographical zones. Retrospective and prospective data will be encoded using a standardized, geography-specific coding system modeled on the *International Classification of Diseases* and SNOMED Clinical Terms (SNOMED CT). Stakeholders, including clinicians, researchers, and public health specialists, will contribute to the development of the coding system using the Delphi methodology. Training will be provided to project assistants for real-time data entry. A snakebite surveillance dashboard will be developed and hosted by the Indian Council of Medical Research.

**Results:**

The study is expected to establish a standardized, geography-specific syndromic coding and reporting system for SBE across selected districts in India. Its implementation is anticipated to improve classification accuracy, reporting completeness, and epidemiological data quality, thereby strengthening surveillance and supporting evidence-based clinical management and policy planning. The project’s funding was approved in January 2025, and participating institutions provided ethics approval between March 2025 and May 2025. The geography-specific syndromic coding framework development, including stakeholder consultations and Delphi rounds, was conducted between June 2025 and August 2025, with prospective implementation of the coding and reporting system planned from September 2025 to September 2026. Data cleaning, validation, and statistical analyses are expected to be completed by March 2027, with the dissemination of findings anticipated in June 2027.

**Conclusions:**

The study proposes a first-of-its-kind phenotypic data–driven approach to snakebite surveillance and treatment in India. The resulting coding system and dashboard will strengthen national reporting and guide future health policy and intervention programs.

## Introduction

Snakebite envenoming (SBE) is a neglected tropical disease of major global health importance, causing significant mortality, morbidity, and socioeconomic consequences, particularly in low- and middle-income countries [1-3]. The World Health Organization (WHO) estimates that snakebites result in 81,000 to 138,000 deaths and 400,000 permanent disabilities annually worldwide [4]. India contributes disproportionately to this burden, accounting for nearly half of global snakebite deaths [1,3,5]. A nationally representative mortality survey reported that approximately 1.2 million snakebite deaths occurred in India between 2000 and 2019, with an annual average of 58,000 deaths, primarily among rural and tribal populations [1,6]. Mortality is highest among young adults and children, underscoring the public health impact on economically productive and vulnerable groups [1,5].

Despite this heavy burden, snakebite remains grossly underreported in official hospital records, leading to significant gaps in surveillance and public health response [6-8]. Only around 30% of people with snakebites reach formal health care facilities, with the majority seeking traditional or informal treatments that often delay lifesaving care [2,9]. This underutilization of health care services, combined with fragmented case reporting and poor medicolegal documentation, has contributed to a chronic underestimation of snakebite mortality and morbidity [6]. The lack of accurate data has further hindered effective planning, allocation of resources, and monitoring of interventions [10-12].

SBE is intricately linked with poverty, agricultural work, and ecological exposure, disproportionately affecting rural communities in South Asia and sub-Saharan Africa.

In India, the highest number of cases and fatalities occur in states such as Uttar Pradesh, Odisha, Madhya Pradesh, and Bihar, largely driven by exposure to the “Big Four” medically important snakes—Russell’s viper, Indian cobra, common krait, and saw-scaled viper [6,13]. However, focusing exclusively on these 4 species overlooks the clinical importance of other venomous snakes in India’s diverse ecological zones [13]. Mapping studies highlight that snakebite risk is unevenly distributed, with hotspots concentrated in tribal and forested areas where access to health services is limited [4,7,14].

The mainstay of snakebite management is the prompt administration of effective antivenom, yet its accessibility and distribution remain suboptimal in many parts of India [2,11,15]. Additional challenges include variation in clinical presentations, the absence of point-of-care diagnostics, and dependence on a syndromic approach to classify envenoming as hemotoxic or neurotoxic based on clinical signs. While practical in resource-limited settings, this approach is inconsistently applied and rarely integrated into structured reporting systems [2,8]. The absence of a standardized diagnostic coding system, coupled with poor interoperability of health information systems, hampers both clinical decision-making and public health planning [6,8,9].

To address these challenges, the WHO launched its global strategy for the prevention and control of snakebite-related mortality and disability by 2030 [12]. India has also developed the National Action Plan for Prevention and Control of Snakebite Envenoming in 2024, which emphasizes surveillance, health system strengthening, and community engagement [16].

However, the success of these strategies depends on accurate data capture, standardized reporting, and multisectoral coordination across health, veterinary, and environmental systems [3,5].

The D-SERPENT initiative (Data-Driven Approaches to Snakebite: Epidemiology, Reporting, Phenotypes, Environmental Factors, Inequalities, and Treatment Duration) seeks to fill this critical gap. It aims to develop and validate an Indian coding system for SBE, aligned with the *International Classification of Diseases* (*ICD*) and SNOMED Clinical Terms (SNOMED CT) principles but adapted to regional syndromic patterns. By applying this coding system prospectively to new cases and retrospectively to datasets such as those generated by the Indian Council of Medical Research (ICMR) task force on snakebite, the initiative will enhance epidemiological surveillance, clinical decision-making, and policy formulation [5,9,17].

Furthermore, adopting a One Health framework that links human health, veterinary services, and environmental surveillance provides an opportunity to address the broader determinants of SBE [3,10,18]. By piloting this approach in high-burden Indian districts, the project will generate evidence on feasibility, cost-effectiveness, and scalability, directly contributing to national and global targets for snakebite control [5,12,19]. The importance of strengthened surveillance systems, standardized reporting mechanisms, and improved data quality for supporting evidence-based snakebite prevention and control strategies has been highlighted through recent policy and research initiatives. Accurate and timely surveillance data have been recognized as essential for the monitoring of disease burden, identification of high-risk populations, evaluation of interventions, and guidance of resource allocation at both national and subnational levels [20]. Limited comparability of snakebite data across health care facilities and geographical regions has been attributed to variations in the clinical classification and documentation of snakebite syndromes. In addition, the integration of snakebite-related information within routine health information systems and surveillance platforms has been hindered by the absence of standardized coding approaches [16,20]. Improvements in data quality, interoperability, disease surveillance, and health system planning have been demonstrated through the use of internationally recognized coding systems and standardized terminologies. Greater consistency and comparability of health information across different settings may also be achieved through the integration of standardized coding frameworks within digital health systems [21-23]. Increasing attention has been directed toward the application of One Health approaches in the prevention and control of SBE through the integration of human, animal, and environmental health sectors. The strengthening of surveillance systems, identification of ecological risk factors, and support of comprehensive public health responses in endemic settings have been facilitated through such approaches [24]. Despite the increasing recognition of SBE as a priority neglected tropical disease, limited evidence has been generated regarding the implementation and evaluation of standardized coding systems specifically designed for snakebite surveillance. Consequently, the development of context-specific reporting frameworks capable of capturing syndromic, epidemiological, and clinical information has been identified as an important public health and research priority [22,23,25].

The primary objective of this study is to develop, validate, and implement a geography-specific syndromic coding and reporting system for SBE to improve standardized documentation, clinical management, and surveillance across selected districts in India.

The secondary objectives are to retrospectively and prospectively evaluate the coding system for coding accuracy, reporting completeness, case classification, and epidemiological data quality, and to assess the applicability of the geography-specific syndromic coding system across selected study districts using retrospective and prospective snakebite data.

## Methods

### Study Design and Setting

This study will adopt an implementation-effectiveness hybrid design, embedded within an observational, single-arm interventional framework, to develop, validate, and evaluate a geography-specific, syndromic coding system for SBE. The study will be conducted in 4 selected districts representing the eastern, western, southern, and northern geographical zones of India. Districts from Odisha, Maharashtra, Kerala, and Himachal Pradesh (Figure 1) have been selected based on the documented burden of SBE and the presence of diverse medically important snake species. All public and private health care facilities involved in snakebite management within the selected districts will be included in the study. This study uses a type 3 implementation-effectiveness hybrid design, with the primary emphasis on implementing a geography-specific syndromic coding framework for SBE. The effectiveness of the coding framework will also be assessed by examining improvements in reporting completeness, standardized documentation, case classification, and epidemiological data quality. As this is a single-arm study, no concurrent control group will be included. Instead, implementation outcomes will be compared with routine reporting practices and retrospectively coded data from the ICMR National Task Force database.

**Figure 1.**
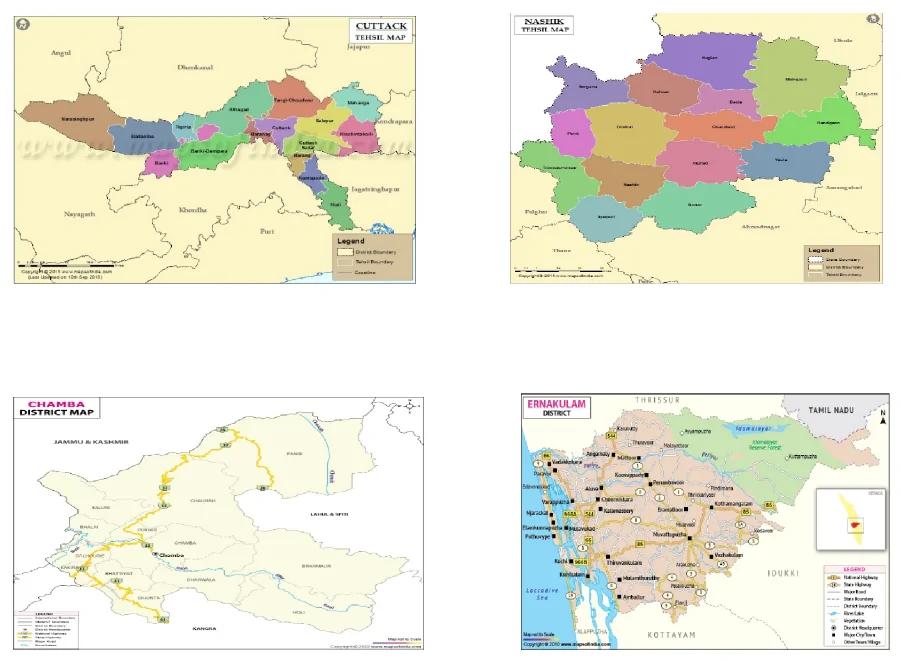
Map of the study setting illustrating 4 selected districts for the implementation-effectiveness hybrid study on snakebite envenoming in India.

The implementation intervention consists of introducing the standardized syndromic coding system, electronic data entry platform, and training of project assistants responsible for prospective data collection and reporting. As this is a single-arm implementation study, no parallel control group will be included. Instead, implementation outcomes will be compared with existing routine reporting practices and retrospective data available from the ICMR National Task Force database.

The study will be conducted in 2 sequential phases. Phase 1 will include the development of the coding framework through literature review, mapping of medically important snake species, stakeholder consultation using a modified Delphi process, and retrospective validation using the ICMR National Task Force database. Phase 2 will involve prospective implementation of the finalized coding system across participating health care facilities with regular monitoring, training, and quality assurance.

The participating health care facilities will serve as the units of implementation, while individual snakebite cases will constitute the units of analysis. Coding system validation will assess coding accuracy, agreement with clinical diagnoses, interrater reliability, and completeness of mandatory coding variables. The effectiveness component of the study will evaluate improvements in reporting completeness, standardized documentation, case classification, and epidemiological data quality following implementation of the coding system.

### Study Participants and Eligibility Criteria

The study will include all individuals with snakebite who present to public or private health care facilities in the selected districts of Odisha, Maharashtra, Kerala, and Himachal Pradesh during the study period (Textbox 1).

Textbox 1. **Inclusion and exclusion criteria.**

**Inclusion criteria**

- All patients presenting with suspected or confirmed snakebite to participating health care facilities during the study period
- Snakebite cases recorded in the Indian Council of Medical Research National Task Force database for retrospective validation

**Exclusion criteria**

- Records with insufficient information to permit coding using the proposed framework
- Patients or legal guardians unwilling to provide informed consent for prospective data collection

Participants for the Delphi process will be selected purposively from among clinicians, nurses, nonphysician health care workers, hospital administrators, public health officials, policymakers, community representatives, and other stakeholders involved in snakebite management, reporting, or surveillance.

### Sample Size

The study will include all individuals with snakebite presenting to any public or private health care facility in the selected study districts during the implementation period. On the basis of available surveillance data, hospital records, and estimates generated from the ICMR National Task Force project, approximately 1000 to 1500 prospective snakebite cases are expected to be captured across the 4 study districts during the 1-year implementation period. In addition, approximately 1000 to 2000 retrospective snakebite records are expected to be available from the ICMR National Task Force database for validation purposes. The inclusion of all eligible snakebite cases is expected to provide sufficient statistical power for assessment of coding accuracy, regional variation in snakebite patterns, reporting completeness, implementation effectiveness, and cost-benefit outcomes.

### Study Procedure

#### Phase 1: Development of the Coding System

Phase 1 will be focused on the development of a geography-specific, syndromic coding system for SBE. Initially, mapping exercises will be undertaken to identify health care facilities involved in snakebite management and to document medically important snake species within each study district. Existing literature, national guidelines, *International Classification of Diseases, Tenth Revision* (*ICD-10*) classifications, SNOMED CT principles, and findings from the ICMR National Task Force project will be reviewed for the development of a preliminary coding framework (Multimedia Appendix 1). The coding framework will be developed in alignment with *ICD-10* and SNOMED CT principles to facilitate future interoperability with existing health information and surveillance systems.

A modified Delphi process was conducted to achieve consensus on the coding framework. A panel of 12 experts—including clinicians, emergency physicians, toxicologists, public health specialists, epidemiologists, nonphysician health care workers, policymakers, and community representatives—was selected purposively based on their expertise in snakebite management, clinical care, public health, research, and snake conservation. Of these, 9 experts participated in all 3 Delphi rounds. The questionnaires were circulated electronically, and responses were collected independently to maintain anonymity. Feedback and suggestions from each round were reviewed and incorporated into the questionnaire for the subsequent round. Consensus was defined as at least 80% agreement among the panel members. Items that did not reach the predefined consensus level were reviewed based on the experts’ comments and were either modified, retained for consideration, or removed. Through successive rounds, the proposed coding structure and list of medically important snake species were refined, and the final coding framework was developed. Validation of the coding framework will be undertaken using both retrospective and prospective snakebite datasets (Figure 1). Coding classifications generated through the framework will be compared with clinical diagnoses, syndromic presentations, treatment records, and expert review, which will serve as the reference standard. Coding accuracy, classification agreement, interrater reliability, and data completeness will be assessed using percentage agreement, Cohen κ statistic, independent coding by trained evaluators, and evaluation of the completeness of mandatory reporting variables. Findings from the validation process will be used for refinement and finalization of the framework prior to wider implementation. Incorporation of syndromic patterns specific to regional snake species will be undertaken within the final coding framework. A menu-driven platform will subsequently be developed to facilitate standardized data entry and reporting. Prior to implementation, training on snakebite syndromes, coding procedures, data collection tools, and quality assurance procedures will be provided to project assistants and research associates.

#### Phase 2: Implementation of the Coding System

During phase 2, the coding system will be prospectively implemented across all participating health care facilities in the selected districts. Data from all eligible snakebite cases will be collected and entered into electronic data collection tools integrated with the coding framework by trained project assistants. Regular monitoring visits, data quality assessments, and review meetings will be conducted throughout the implementation period. These activities will involve the project assistants, coprincipal investigators, and the principal investigator to identify implementation challenges and support the iterative refinement of the coding system. Implementation effectiveness will be assessed by evaluating reporting completeness, standardized documentation, adoption of the coding framework across participating health care facilities, and improvements in the quality and consistency of epidemiological data generated during routine clinical practice. Monitoring findings and stakeholder feedback will also be used to identify implementation challenges and refine the coding system during the study. During phase 2, the finalized coding system will be implemented prospectively across the participating health care facilities in the selected districts. All eligible snakebite cases will be recorded using electronic data collection tools incorporating the standardized coding framework. Prior to implementation, training on snakebite syndromes, coding procedures, data collection, and quality assurance will be provided to project assistants and research associates. Regular monitoring visits, data quality assessments, and review meetings will be conducted throughout the implementation period. The feasibility and acceptability of the coding framework will be assessed based on its applicability to routine case documentation, completeness of required reporting fields, consistency of case classification, and utility for standardized epidemiological reporting and surveillance.

### Retrospective Validation of the Coding System

The coding framework will be retrospectively applied to snakebite records available in the ICMR National Task Force project database. Validation will be undertaken against a reference standard comprising documented clinical diagnoses, syndromic classifications, laboratory findings, treatment records, and independent expert review. Coding accuracy will be assessed as the proportion of cases correctly classified by the coding framework in comparison with the reference standard. Classification agreement between the coding framework and clinician-assigned diagnoses will be evaluated. Interrater reliability will be assessed through independent coding of a subset of records by 2 trained coders. Agreement between coders will be quantified using Cohen κ statistic. Interpretation of κ values will be performed according to established benchmarks for agreement. Data completeness will be evaluated by calculating the proportion of mandatory coding fields completed for each record. Completeness rates will be reported overall and by district, and patterns of missing data will be examined to identify areas requiring further refinement of the coding system.

### Statistical Analysis

Descriptive and comparative analyses will be conducted to evaluate the performance and applicability of the geography-specific syndromic coding system for SBE (Multimedia Appendix 2). Reporting completeness and the effectiveness of the coding framework will be assessed through calculation of the proportion of snakebite cases treated at participating health care facilities. Demographic and clinical characteristics of people with snakebites, including age, sex, geographical location, syndromic classification, presumptive snake species, treatment received, and clinical outcomes, will be summarized using frequencies and percentages for categorical variables and means with SDs or medians with IQRs for continuous variables, as appropriate. Coding accuracy will be evaluated through comparison of coded classifications with clinical diagnoses and syndromic assessments documented in patient records. Interrater reliability will be assessed using a subset of records independently coded by 2 trained coders, and agreement will be quantified using Cohen κ statistic. Data completeness will be evaluated by determining the proportion of mandatory coding fields completed within each record. Hospital stay duration, treatment patterns, complications, and clinical outcomes will be described according to syndromic classification and geographical region. Coding system validation and implementation effectiveness will be evaluated separately. Validation outcomes will include coding accuracy, agreement with the reference standard, interrater reliability, and completeness of mandatory coding variables. Effectiveness outcomes will include improvements in reporting completeness, standardized documentation, case classification, and epidemiological data quality following implementation of the coding system. For the implementation evaluation, these outcomes will be compared between the preimplementation and postimplementation periods. Appropriate regression models will be applied according to the type of outcome, with clustering at the health care facility and district levels accounted for using mixed-effects models or cluster-robust standard errors, where appropriate. The extent and pattern of missing data will be assessed, and complete-case analysis will be undertaken where the proportion of missing observations is limited. Sensitivity analyses will be undertaken by study district and type of health care facility, where sufficient observations are available, and temporal trends will be considered to account for potential secular changes in reporting. Historical data from the ICMR National Task Force database will be used as a reference for retrospective validation and comparison with routine reporting rather than as a concurrent control group. Differences in population characteristics, health care settings, study periods, and case ascertainment will be considered when interpreting these comparisons. Where multiple comparisons are undertaken, findings will be interpreted with consideration of effect estimates and 95% CIs. The utility of the coding framework for epidemiological reporting, surveillance, and potential integration into national snakebite reporting systems will be assessed using findings generated from both prospective and retrospective analyses. All statistical analyses will be performed using appropriate statistical software. A *P* value of <.05 will be considered statistically significant wherever inferential statistical testing is undertaken.

### Cost-Benefit Analysis

A cost-benefit analysis will be conducted from the health system perspective. Costs associated with software development, dashboard maintenance, personnel, training activities, monitoring, and data management will be identified, documented, and quantified throughout the study period. Benefits arising from the implementation of the coding framework will be assessed in terms of improvements in reporting completeness, coding accuracy, surveillance utility, and availability of data for public health planning and policy development. The relationship between implementation costs and reporting outcomes will be evaluated, and the incremental cost per additional correctly reported snakebite case will be estimated.

### Ethical Considerations

Ethics approval for the study has been obtained from the Institutional Ethics Committee of Amrita Institute of Medical Sciences (ECASM-2024‐577), Kochi, ICMR- National Institute for Research in Reproductive and Child Health and ICMR-Regional Medical Research Centre, Bhubaneswar. Administrative approvals will also be secured from the state health departments and relevant district authorities in the selected states prior to the start of data collection. For prospective data collection, written informed consent will be obtained from all participants or their legally authorized representatives. For participants below 18 years of age, parental or guardian consent, together with age-appropriate assent, will be obtained. In cases of acute life-threatening snakebite where immediate consent cannot be provided, deferred consent will subsequently be obtained from the participant or legally authorized representative when clinically feasible. For retrospective record review, a waiver of informed consent will be sought from the relevant ethics committees, as only deidentified secondary data will be used and no direct participant contact will be involved. All collected data will be deidentified prior to analysis, and unique study identification numbers will be assigned to each participant. Access to identifiable information will be restricted to authorized study personnel. Electronic data will be stored on password-protected servers and protected through a predefined governance framework. Role-based access mechanisms will be implemented, and access to district-level or individual-level data will be restricted to authorized users. All data sharing and access procedures will be governed by applicable institutional and national data protection regulations.

## Results

A geography-specific, syndromic coding framework for SBE is expected to be developed, and evidence of its applicability for improving snakebite reporting, classification, and surveillance is expected to be generated to support future integration into public health reporting systems. Funding for the project was approved in January 2025, and ethical approvals were obtained from the participating institutions between March 2025 and May 2025. Development of the geography-specific syndromic coding framework, including stakeholder consultations and Delphi rounds, was scheduled between June 2025 and August 2025. Prospective implementation of the coding and reporting system was planned from September 2025 to September 2026 across the selected districts of Odisha, Maharashtra, Kerala, and Himachal Pradesh. Retrospective validation using data from the ICMR National Task Force project was planned during the implementation phase. Data cleaning, validation, and statistical analyses are expected to be completed by March 2027, and dissemination of findings through scientific publications and stakeholder reports is anticipated in June 2027 (Figure 2).

**Figure 2.**
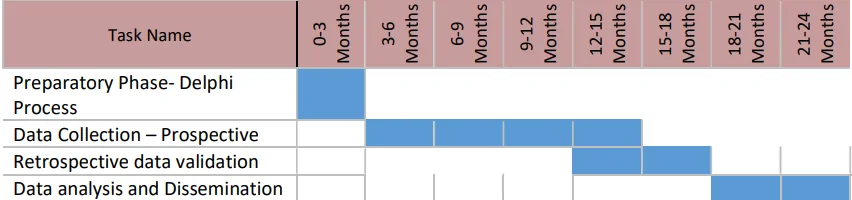
Study timeline for the proposed research.

The study is currently being conducted according to protocol version 2.0; no further protocol amendments have been made. The validation outcomes, including coding accuracy, agreement with the reference standard, interrater reliability, and data completeness, as well as the implementation-effectiveness outcomes, including reporting completeness, standardized documentation, case classification, and the utility of the coding framework for epidemiological surveillance, were predefined before examination of the prospective study data. Data collection is ongoing, and the final analyses will be conducted after completion of the study in accordance with the approved protocol. The *ICD* coding framework is also being validated using a dataset comprising more than 18,000 people with snakebites enrolled in the ICMR National Task Force on the incidence, mortality, morbidity, and socioeconomic burden of snakebite.

## Discussion

### Principal Findings

The development and validation of a geography-specific, syndromic coding framework for SBE are expected to be accomplished through this study. Improvements in the completeness, consistency, and standardization of snakebite reporting across geographically diverse regions of India are anticipated. Enhanced classification of snakebite syndromes and more comprehensive capture of epidemiological information are expected to be achieved through implementation of the proposed coding framework. Evidence regarding the feasibility, applicability, and utility of incorporating a syndromic coding approach into routine snakebite surveillance systems is expected to be generated through prospective implementation and retrospective validation.

### Comparison With Prior Work

Snakebite surveillance in India has traditionally been conducted through hospital-based reporting systems and broad disease classification frameworks, within which detailed information on syndromic presentation, presumptive snake species, treatment patterns, and clinical outcomes has often not been adequately captured. Standardized coding approaches have been provided through international classification systems such as *ICD* and SNOMED CT; however, their applicability to the diverse clinical and ecological settings of SBE in India has not been fully established. To address these limitations, a geography-specific coding framework has been developed through the incorporation of regionally relevant syndromic patterns and epidemiological characteristics. Through the present study, evidence regarding the potential of this framework to complement existing reporting systems and strengthen snakebite surveillance is expected to be generated.

### Strengths and Limitations

Several strengths are expected to be associated with this study. Evaluation of the coding framework in diverse ecological and health system settings will be facilitated through implementation across 4 geographically distinct regions of India. Improved representativeness and broader applicability are expected to be achieved through the inclusion of both public and private health care facilities. The relevance, acceptability, and contextual appropriateness of the coding framework are also expected to be strengthened through the use of stakeholder engagement and Delphi-based consensus procedures [26,27]. Certain limitations are also anticipated. Variations in health care infrastructure, clinical documentation practices, and reporting capacities across study sites may be encountered and may influence implementation. Dependence on retrospective records for validation may result in variability in data completeness and quality. Furthermore, definitive confirmation of snake species may not be available for all cases; consequently, reliance on syndromic classification and clinical assessment may be required in some instances.

### Future Directions

If feasibility and effectiveness are demonstrated, support for the development of a standardized national reporting system for SBE may be provided through the proposed coding framework. Future integration with existing surveillance platforms and health information systems may be facilitated. The evaluation of implementation outcomes and adaptation strategies may provide valuable insights for wider scale-up and integration within routine health systems [28-30]. A foundation for improved epidemiological monitoring, resource allocation, policy planning, and evaluation of snakebite control programs in India may also be established through implementation of the framework.

The findings generated through the study will be disseminated through peer-reviewed scientific publications, conference presentations, technical reports, and stakeholder consultations. Results will be communicated to participating institutions, state health departments, policymakers, and public health authorities to facilitate evidence-informed decision-making. The potential scale-up and integration of the coding framework within routine surveillance systems will also be informed through dissemination of the study findings.

### Anticipated Limitations and Implementation Challenges

Variations in health care infrastructure, documentation practices, and reporting capacities across study sites may influence implementation of the coding framework. Differences between public and private health care facilities may affect data completeness and consistency. Definitive snake species identification may not be available for all cases, requiring reliance on syndromic classification. Variability in the quality of retrospective records and operational challenges related to training, data entry, and integration into existing reporting systems may also be encountered. These challenges will be addressed through regular monitoring, stakeholder engagement, refresher training, and quality assurance measures.

## Supplementary material

10.2196/98861 Multimedia Appendix 1 Syndromic approach.

10.2196/98861 Multimedia Appendix 2 *International Classification of Diseases* (*ICD*) codes.

## Abbreviations

D-SERPENT: Data-Driven Approaches to Snakebite: Epidemiology, Reporting, Phenotypes, Environmental Factors, Inequalities, and Treatment Duration
*ICD*: *International Classification of Diseases*
*ICD-10*: *International Classification of Diseases, Tenth Revision*
ICMR: Indian Council of Medical Research
SBE: snakebite envenoming
SNOMED CT: SNOMED Clinical Terms
WHO: World Health Organization

## References

[1] Mohapatra B, Warrell DA, Suraweera W (2011). Snakebite mortality in India: a nationally representative mortality survey. PLoS Negl Trop Dis.

[2] (2016). Guidelines for the management of snakebites, 2nd edition. World Health Organization.

[3] Kasturiratne A, Wickremasinghe AR, de Silva N (2008). The global burden of snakebite: a literature analysis and modelling based on regional estimates of envenoming and deaths. PLoS Med.

[4] Rahman R, Faiz MA, Selim S (2010). Annual incidence of snake bite in rural Bangladesh. PLOS Negl Trop Dis.

[5] Vaiyapuri S, Vaiyapuri R, Ashokan R (2013). Snakebite and its socio-economic impact on the rural population of Tamil Nadu, India. PLoS One.

[6] Chakma JK, Menon JC, Dhaliwal RS, Indian Council of Medical Research (2020). White paper on venomous snakebite in India. Indian J Med Res.

[7] Suraweera W, Warrell D, Whitaker R (2020). Trends in snakebite deaths in India from 2000 to 2019 in a nationally representative mortality study. Elife.

[8] Longbottom J, Shearer FM, Devine M (2018). Vulnerability to snakebite envenoming: a global mapping of hotspots. Lancet.

[9] Harrison RA, Hargreaves A, Wagstaff SC, Faragher B, Lalloo DG (2009). Snake envenoming: a disease of poverty. PLoS Negl Trop Dis.

[10] Gutiérrez JM, Warrell DA, Williams DJ (2013). The need for full integration of snakebite envenoming within a global strategy to combat the neglected tropical diseases: the way forward. PLoS Negl Trop Dis.

[11] Habib AG, Kuznik A, Hamza M (2015). Snakebite is under appreciated: appraisal of burden from West Africa. PLoS Negl Trop Dis.

[12] (2019). Snakebite envenoming -- a strategy for prevention and control. World Health Organization.

[13] Menon JC, Sreekrishnan TP, Nair SB (2025). Snakebite envenoming in India: it is time we look beyond the concept of the Big Four species. Trans R Soc Trop Med Hyg.

[14] Sharma SK, Chappuis F, Jha N, Bovier PA, Loutan L, Koirala S (2004). Impact of snake bites and determinants of fatal outcomes in southeastern Nepal. Am J Trop Med Hyg.

[15] Alirol E, Sharma SK, Bawaskar HS, Kuch U, Chappuis F (2010). Snake bite in South Asia: a review. PLoS Negl Trop Dis.

[16] (2025). National action plan for prevention and control of snakebite envenoming (NAPSE). Ministry of Health and Family Welfare, Government of India.

[17] Williams DJ, Faiz MA, Abela-Ridder B (2019). Strategy for a globally coordinated response to a priority neglected tropical disease: snakebite envenoming. PLoS Negl Trop Dis.

[18] Chippaux JP (2017). Snakebite envenomation turns again into a neglected tropical disease!. J Venom Anim Toxins Incl Trop Dis.

[19] Ralph R, Sharma SK, Faiz MA (2019). The timing is right to end snakebite deaths in South Asia. BMJ.

[20] (2023). Regional action plan for prevention and control of snakebite envenoming in the South-East Asia 2022–2030. World Health Organization.

[21] (2021). Global strategy on digital health 2020-2025. World Health Organization.

[22] International Classification of Diseases 11th revision. World Health Organization.

[23] SNOMED CT starter guide. SNOMED International.

[24] (2022). One Health Joint Plan of Action (‎2022‒2026)‎: working together for the health of humans, animals, plants and the environment. World Health Organization.

[25] Gutiérrez JM, Calvete JJ, Habib AG, Harrison RA, Williams DJ, Warrell DA (2017). Snakebite envenoming. Nat Rev Dis Primers.

[26] Hasson F, Keeney S, McKenna H (2000). Research guidelines for the Delphi survey technique. J Adv Nurs.

[27] Keeney S, Hasson F, McKenna HP (2011). The Delphi Technique in Nursing and Health Research.

[28] Proctor E, Silmere H, Raghavan R (2011). Outcomes for implementation research: conceptual distinctions, measurement challenges, and research agenda. Adm Policy Ment Health.

[29] Damschroder LJ, Aron DC, Keith RE, Kirsh SR, Alexander JA, Lowery JC (2009). Fostering implementation of health services research findings into practice: a consolidated framework for advancing implementation science. Implement Sci.

[30] Powell BJ, Waltz TJ, Chinman MJ (2015). A refined compilation of implementation strategies: results from the Expert Recommendations for Implementing Change (ERIC) project. Implement Sci.

