# Supplementary material for: Development, Validation, and Implementation of a Geography-Specific Syndromic Coding and Reporting System for Snakebite Envenoming (D-SERPENT): Protocol for an Implementation-Effectiveness Hybrid Study
Source: JMIR Res Protoc. 2026 Nov 10;15:e98861. doi: 10.2196/98861 (PMC13750521; doi:10.2196/98861)
Supplement: Supplementary file 1 [file resprot-v15-e98861-s001.docx]

Figure-1:

Unknown Snakebite

Neurotoxic Symptoms

Local Inflammation at bite site

Yes

No Bleeding

Cobra Bite

King Cobra

Cobra

No

Bitten on Land

Krait Bite

Common krait

Bitten while at sea

Sea Snake

Cochin banded sea snake

Hemotoxic Symptoms

Predominant Localized Signs

other viper species-HNPV/MPV

Hump nosed pit viper

Malabar pit viper

Saw Scaled Viper

Severe Inflammation AKI, Low Platelets

Russell's Viper

KERALA- ERNAKULAM

Figure-2:

Unknown Snakebite

Neurotoxic Symptoms

Inflammation

Yes

No Bleeding

Cobra Bite

King Cobra

Monocellate cobra

Cobra

No

Bitten on Land

Krait Bite

Common Krait

Bitten while at sea

Sea Snake

Beaked Sea Snake

Hemotoxic Symptoms

Predominant Localized Signs

Saw Scaled Viper

Severe Inflammation AKI, Low Platlets

Russell's Viper

ODISHA- CUTTACK

Figure-3:

Unknown Snakebite

Neurotoxic Symptoms

Inflammation

Yes

No Bleeding

Cobra Bite

Black cobra

Central Asian Cobra

No

Bitten on Land

Krait Bite

Coral Bite

Hemotoxic Symptoms

Predominant Localized Signs

other viper species

Himalayan pitviper

Western green pitviper

Severe Inflammation AKI, Low Platlets

Russell's Viper

HIMACHAL PRADESH- CHAMMBA

Figure-4:

Unknown Snakebite

Neurotoxic Symptoms

Inflammation

Yes

No Bleeding

Cobra Bite

Binocellate Cobra,

No

Bitten on Land

Krait Bite

Common Krait

Coral bite

Slender Coral snake

Kahndala Coral Snake(

Hemotoxic Symptoms

Predominant Localized Signs

Saw Scaled Viper

Bamboo Pit Viper

Severe Inflammation AKI, Low Platlets

Russel Viper

MAHARASHTRA - NASIK
