# Supplementary material for: Development, Validation, and Implementation of a Geography-Specific Syndromic Coding and Reporting System for Snakebite Envenoming (D-SERPENT): Protocol for an Implementation-Effectiveness Hybrid Study
Source: JMIR Res Protoc. 2026 Nov 10;15:e98861. doi: 10.2196/98861 (PMC13750521; doi:10.2196/98861)
Supplement: Supplementary file 2 [file resprot-v15-e98861-s002.docx]

ICD codes

 *Viper - XE8LD*

1. Russells Viper : **XE8HR** (hemotoxic russells)
2. Saw Scale Viper : **XE8SE** (Saw scale Ecarin)
3. Sochurek's Saw Scale Viper : **XE8SR**(Sochurek-Renal failure)
4. HNPV : **XE8HH**  ( Hemotoxic Hypnale)
5. Himalayan Pit Viper: **XE8HG** (Hemotoxic Gloydius)
6. Chamba Pit Viper : **XE8HC** (Hemotoxic Chambensis)
7. Northern White Lipped Pit Viper : **XE8TS** (Trimeresurus septentrionalis)
8. Bamboo Pit Viper : **XE8CG** (Craspedocephalus Gramineus)
9. Salazar Pit Viper : **XE8HS** (Hemotoxic Salazar)
10. Large Scaled Pit Viper: **XE8CP** (Craspedocephalus Peltopelor)
11. Horse Shoe Pit Viper : **XE8CS** (Craspedocephalus Strigatus)

 *Cobra - XE9X2*

1.  Indian Cobra : **XE9NP** (Neurotoxic Postsynaptic Naja)
2. Caspian Cobra/Black Cobra : **XE9CN** ( Central Asian Neurotoxic Naja)
3. Monocled Cobra : **XE9MC** ( Monocled Cobratoxin)  

 *Krait - XE2UZ*

1. Common krait : **XE2PB** ( Presynaptic Bungarotoxin)
2. Banded Krait : **XE2PF** (Presynaptic Fasciatus)
3. Sind Krait : **XE2SB** (Sind Bungarotoxin)
4. Greater Black Krait : **XE2NN** (Neurotoxic niger)
5. Himalayan krait : **XE2BB** (Bungaroides Bungarotoxin)
6. Lesser Black Krait : **XE2LB**   (Lividus Bungarotoxin)
7. Wall's Krait : **XE2WN** (Walli's Neurotoxin)
8. Suzhen Krait : **XE2PS** (Presynaptic Suzhenae

 *King Cobra*
 
1. Kaalinga : **XE0NK** (Neurotoxic Kaalinga KingCobra)
2. Hannah : **XE0HN** (Neurotoxic Hannah)

 *Indian Coral Snakes*

1. Slender Coral snake : **XE3CM** (Calliophis Melanurus)
2. Striped Coral Snake: **XE3NN** (Neurotoxic Nigrescence)
3. Bibrons Coral Snake : **XE3CB** (Calliophis Bibroni)
4. Beddomes Coral Snake : **XE3NB** ( Neurotoxic Beddomei)
5. Khandala Coral Snake : **XE3NK** (Nigrescence Khandalensis)
6. MacClellands Coral Snake : **XE3SM** (Sinomicrurus Macclellandii)

 *Keelback*
 
Red Necked Keelback : **XE7RC** ( Rabdophis Coagulopathy envenomation)

 *Sea Snakes - **XE48L***

1. Hook Nosed Sea Snake : **XE48S**    (Schistosus)
2. Annulated : **XE48C**   (Cyanocinctus)
3. Cochin Banded : **XE48G**   (Gray's)
4. Yellow : **XE48P**   (Platurus)
5. Short Sea Snake : **XE48A**  (Apraefrontalis)
6. Yellow Lipped Sea Krait : **XE48B**  (Banded ) XE2LB
